# Supplementary material for: Fermented cereal-based Munkoyo beverage: Processing practices, microbial diversity and aroma compounds
Source: PLoS One. 2019 Oct 22;14(10):e0223501. doi: 10.1371/journal.pone.0223501 (PMC6805097; doi:10.1371/journal.pone.0223501)
Supplement: S1 Questionnaire — (DOCX) [file pone.0223501.s001.docx]

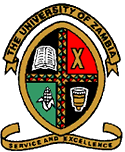

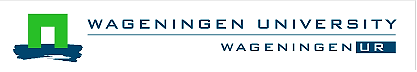


**Questionnaire for Key Informants on the Different Methods of Processing Munkoyo**

Dear Respondent,

I am ………………………………………………………………………, an enumerator for the munkoyo processing survey. This survey aims at collecting indigenous knowledge on different ways of producing munkoyo and the factors that influence its quality with a view of improving and optimizing its production at different scales for local communities. This study is part of a sandwich PhD research project under the University of Zambia in collaboration with Wageningen University of The Netherlands being carried out by Sydney Phiri. We thank you for your participation.

**Questionnaire No. ………….**

1. **Questionnaire administration information**

| **N°** | **Questions** | **Answers** | **Code** |
| --- | --- | --- | --- |
| 1.1 | Name of enumerator |  |  |
| 1.2 | Name of respondent |  |  |
| 1.3 | Date of interview |  |  |
| 1.4 | Time | Start :  Finish : |  |
| 1.5 | Location ( coordinates) | Village....................................................................  Camp.....................................................................  Block...................................................................  District.................................................................  Province............................................................... |  |
| 1.6 | Contact Number (Mobile phone) |  |  |
| 1.7 | Main language used by respondent | 1. English  2. Tonga  3. Nyanja  4. Bemba  5. Other (specify)............................................... |  |
| 1.8 | Was a translater used? | 1. Yes 2. No |  |
| 1.9 | What activities are you involved in with respect to munkoyo? | 1. Trader 2. Processor 3. Consumer |  |

1. **Socio- demographic information**

| **N°** | **Questions** | **Answers** | **Code** |
| --- | --- | --- | --- |
| 2.1 | Gender | 1. Male  2. Female |  |
| 2.2 | Ethnic group (Tribe) | 1. Lozi 2. Tonga 3. Nyanja 4. Bemba 5. Lunda 6. Kaonde 7. Luvale 8. Other (specify)................................................... |  |
| 2.3 | Marital status | 1. Single  2. Married  3. Divorced  4. Widow/ widower |  |
| 2.4 | Age in years | 1. ≤18  2. 19-29  3. 30-39  4. 40-49  5. 50-59  6. 60+ |  |
| 2.5 | Education level | 1. No education  2. Primary (Grade 7)  3. Secondary (O-Level or A-Level)  4. Vocational training  5. Tertiary (diploma or degree)  6. Other (specify)......................................................................... |  |
| 2.6 | Who is head of the household? | 1. Father 2. Mother 3. Child 4. Grandmother 5. Grandfather 6. Aunt 7. Uncle 8. Other (specify)....................................................................... |  |
| 2.7 | Size of the Household | 1. 0-6 2. 6-10 3. >10 4. Other (specify) …………………………………………………………………. |  |

1. **Munkoyo processing methods**

| **N°** | **Questions** | **Answers** | **Code** |
| --- | --- | --- | --- |
| 3.1 | How long do you cook the porridge before adding the roots? | 1. 30 minutes  2. One hour   1. 0ne and half hours 2. Two hours   5. Until it start burning |  |
| 3.2 | How hot is the porridge before adding the roots? | 1. Not too hot to burn you (50 – 55^o^C)  2. Able to feel the warmth on the arm (40 – 45 ^o^C)  3. Just cold (25- 35^o^C)  4. It does not matter |  |
| 3.3 | How much root do you / can you add to produce 20L munkoyo? | 1. 3 strips of roots  2. 5 strips of roots  3. 7 strips of roots  4. 10 strips of roots |  |
| 3.4 | For how long do you leave the strips of roots in the cooked porridge? | 1. One hour 2. Five hours 3. One day 4. Until munkoyo is consumed. |  |
| 3.5 | Can you reuse the roots? | 1. Yes **(Two times) (Three times) (Many times**) 2. No |  |
| 3.6 | Is the quality of munkoyo different if you use something else other than munkoyo roots? | 1. Yes **(Taste is different) (Flavour is different) (Bad appearance)**  2. No |  |
| 3.7 | Can you process munkoyo without munkoyo roots? | 1. Yes You can use **(Wheat flour) (Malted millet or sorghum)**  2. No |  |
| 3.8 | What containers do you use to make Munkoyo? | 1. Plastic bucket  2. Metal pot  3. Calabash (Nsuwa)  4. Earthen pot (Nongo) |  |
| 3.9 | Where do you ferment/incubate your munkoyo? | 1. In the house  2. Outside in the sun  3. Outside in the shed |  |
| 3.10 | How long do you ferment munkoyo before selling or Consumption? | 1. Half a day (12hrs)  2. One day  3. Two days  4. Three days |  |

1. **Consumer perception on quality of munkoyo**

| **N°** | **Questions** | **Answers** | **Code** |
| --- | --- | --- | --- |
| 4.1 | What determines the quality of Munkoyo? | 1. Good appearance  2. Good taste  3. Good flavour  4. All of the above |  |
| 4.2 | How do you consume munkoyo? | 1. As breakfast 2. As Lunch 3. As a social drink 4. As energy drink |  |
| 4.3 | When do you usually consume Munkoyo? | 1. When you are hungry 2. Before a meal 3. After a meal 4. When relaxing |  |
| 4.4 | What do you like about munkoyo as a beverage? | 1. Good flavour  2. Good taste  3. Good colour  4. Grits |  |
| 4.5 | Where do you think munkoyo is much more consumed? | 1. Social gatherings **(funerals) (Weddings)** 2. Household level |  |
| 4.6 | Who likes consuming munkoyo? | 1. Women 2. Men 3. Girls 4. Boys 5. Everyone |  |
| 4.7 | What do you think can be done to improve the consumption of Munkoyo? | 1. Package it 2. Sensitizing the value of munkoyo 3. Encourage house hold processing |  |
| 4.8 | What is the most recommended food for a weaned baby? | 1. Porridge with milk 2. Porridge with groundnuts |  |
| 4.8 | Can you give munkoyo to a weaned baby | 1. Yes 2. No! Why? **(Baby does not like it) (Causes Diarrhoea)** |  |
| 4.9 | What kind of the drink would you prefer to give a weaned baby? | 1. Munkoyo  2. Mabisi |  |
| 4.10 | Why do you prefer Munkoyo or mabisi you have chosen above? | 1. Good taste  2. Good flavour  3. High in nutrients  4. That’s what is available |  |
| 4.11 | Would you recommend munkoyo as a weaning food as exclusively done with breast milk? | 1. Yes  2. No Why?**(Fermented food not good for the baby) (No nutrients in munkoyo like that in breast milk)) (Usually contaminated by bacteria)** |  |
| 4.12 | Do you think it can be a good idea to process munkoyo with groundnuts? | 1. Yes  2. No |  |
| 4.13 | What do you think can be the value of adding groundnuts in the processing of munkoyo? | 1. Can add protein and nutrients  2. Can add good taste  3. Can add good flavour  4. Nothing special |  |
